# Patient Experiences Navigating Care Coordination For Long COVID: A Qualitative Study

**DOI:** 10.1007/s11606-024-08622-z

**Published:** 2024-02-02

**Authors:** Sarah R. MacEwan, Saurabh Rahurkar, Willi L. Tarver, Cortney Forward, Jennifer L. Eramo, Lauren Teuschler, Alice A. Gaughan, Laura J. Rush, Stacy Stanwick, Erin McConnell, Andrew Schamess, Ann Scheck McAlearney

**Affiliations:** 1 Division of General Internal Medicine, College of Medicine, The Ohio State University, Columbus, OH USA; 2 Center for the Advancement of Team Science, Analytics, and Systems Thinking in Health Services and Implementation Science Research (CATALYST), College of Medicine, The Ohio State University, Columbus, OH USA; 3 Department of Biomedical Informatics, College of Medicine, The Ohio State University, Columbus, OH USA; 4 Division of Cancer Prevention and Control, The Ohio State University, Columbus, OH USA; 5 Department of Family and Community Medicine, College of Medicine, The Ohio State University, Columbus, OH USA

**Keywords:** post-acute COVID-19 syndrome, long COVID, care coordination, chronic disease, qualitative research

## Abstract

**Background:**

Little is known about how to best evaluate, diagnose, and treat long COVID, which presents challenges for patients as they seek care.

**Objective:**

Understand experiences of patients as they navigate care for long COVID.

**Design:**

Qualitative study involving interviews with patients about topics related to seeking and receiving care for long COVID.

**Participants:**

Eligible patients were at least 18 years of age, spoke English, self-identified as functioning well prior to COVID infection, and reported long COVID symptoms continued to impact their lives at 3 months or more after a COVID infection.

**Approach:**

Patients were recruited from a post-COVID recovery clinic at an academic medical center from August to September 2022. Interviews were audio-recorded, transcribed, and analyzed using thematic analysis.

**Key Results:**

Participants (*n*=21) reported experiences related to elements of care coordination: access to care, evaluation, treatment, and ongoing care concerns. Some patients noted access to care was facilitated by having providers that listened to and validated their symptoms; other patients reported feeling their access to care was hindered by providers who did not believe or understand their symptoms. Patients reported confusion around how to communicate their symptoms when being evaluated for long COVID, and they expressed frustration with receiving test results that were normal or diagnoses that were not directly attributed to long COVID. Patients acknowledged that clinicians are still learning how to treat long COVID, and they voiced appreciation for providers who are willing to try new treatment approaches. Patients expressed ongoing care concerns, including feeling there is nothing more that can be done, and questioned long-term impacts on their aging and life expectancy.

**Conclusions:**

Our findings shed light on challenges faced by patients with long COVID as they seek care. Healthcare systems and providers should consider these challenges when developing strategies to improve care coordination for patients with long COVID.

**Supplementary Information:**

The online version contains supplementary material available at 10.1007/s11606-024-08622-z.

## INTRODUCTION

It has been estimated that 65–200 million individuals worldwide have been afflicted with long COVID.^1,2^ Patients with long COVID experience prolonged symptoms following acute infection including post-exertional malaise, cognitive impairment, fatigue, palpitations, headache, depression or anxiety, loss of taste or smell, and shortness of breath.^3–5^ These symptoms can range from mild to debilitating, and they may last weeks, months, or years. Despite ongoing research into the biological basis of long COVID, there have been very few clinical trials of potential therapies to date.^1,6,7^ Because of this, evidence-based guidelines to treat long COVID are lacking. Although attempts have been made to summarize clinical opinion and available evidence, this information is not widely known or implemented.^8–17^

Long COVID meets several criteria for the World Health Organization’s definition of a chronic disease: it results from non-reversible pathological alterations, may require a period of rehabilitation, and may require ongoing management over a period of years.^18–20^ Typically, patients suffering from chronic conditions, such as diabetes, hypertension, or human immunodeficiency virus, seek care from multiple providers across different specialties and care settings. Coordination of care across these disparate providers and settings is critical for successful management and includes initial health care visits, assessment of symptoms, selection and adjustment of treatments, and follow-up, laid out in the chronic care model.^21,22^ Yet little is known about the experiences of patients as they navigate this pathway to seek care for long COVID. In order to fill this gap in knowledge, this study aimed to understand the perspectives of patients with long COVID, framed by consideration of the elements of care coordination.^23^

## METHODS

### Study Population and Setting

This qualitative study involved one-on-one interviews with patients seeking care at a post-COVID recovery clinic within the Ohio State University Wexner Medical Center (OSUWMC). Patients require a physician referral from within OSUWMC to be seen at the post-COVID recovery clinic, which is staffed with general internal medicine physicians who perform initial evaluations and make specialty referrals, most commonly to physical therapy, rehabilitation psychology, and speech therapy. A nurse care coordinator assists patients as they navigate through referrals and follow-up appointments. Eligible patients had to be ≥18 years of age, be English speakers, self-identify as functioning well in daily life before getting COVID, and report long COVID was having a significant impact on their life ≥3 months after a COVID infection. OSU’s Institutional Review Board approved this study.

### Data Collection

Interviews were conducted by five study team members between August and September 2022. Interviewers were PhD- or MS-trained health services researchers and were not practicing clinicians. Purposeful sampling was used to recruit patients based on study eligibility criteria. Post-COVID recovery clinic staff identified eligible patients; introduced the study; and for interested patients, sent contact information to the research team. Research team members then sent emails to interested patients to schedule an interview. Interviews were conducted by phone or video conference. Participants provided verbal informed consent.

A semi-structured interview guide was used to explore perspectives, including those about seeking and receiving care for long COVID (Text Box). The interview guide was developed by members of the research team, including health services researchers and clinicians within the post-COVID recovery clinic. Interviews were audio recorded, transcribed verbatim, and de-identified. Patients were emailed a $25 Amazon gift card in appreciation for their participation.

**Table:** Text Box. Interview questions informing the understanding of perspectives about care coordination for long COVID

Could you please describe your journey with COVID-19 and how you came to receive treatment at the post-COVID recovery clinic?
What treatments have you received for your long COVID symptoms? How have you felt that your healthcare providers outside of the post-COVID recovery clinic have been supportive during your journey with long COVID? In what ways do you feel you are missing this type of support? What do you want doctors to know about what it is like to be a patient with long COVID and how can they make your journey less challenging? What are your greatest concerns about long COVID?

### Data Analysis

Interview transcripts were analyzed using deductive dominant thematic analysis.^24^ A preliminary coding dictionary was created with codes corresponding to questions posed in the semi-structured interview guide. Four members of the research team who were also interviewers coded one transcript using this preliminary coding dictionary. Through weekly group discussions, changes to the coding dictionary were made, including adding emergent codes. These discussions also served to establish a clear understanding of the definition and application of codes. The remaining transcripts were divided among the four research team members who applied codes according to this revised preliminary dictionary. Weekly meetings were used to discuss uncertainty in the application of codes and to ensure consistency of coding across all transcripts.

Data identified with codes related to treatment, healthcare provider support, what participants want providers to know about what it is like being a patient with long COVID, and concerns surrounding long COVID were selected for secondary analysis. In this process, we used inductive thematic analysis to identify and characterize emergent themes within the data that mapped to elements of care coordination^23^ most relevant to patients with long COVID: access to care, evaluation, treatment, and ongoing care concerns. All four research team members who participated in the preliminary coding discussed emergent themes in this secondary analysis process, with one team member applying the new codes to the selected data. ATLAS.ti qualitative analysis software (ATLAS.ti Scientific Software Development GmbH) was used to support these analyses.

## RESULTS

**Figure 1:**
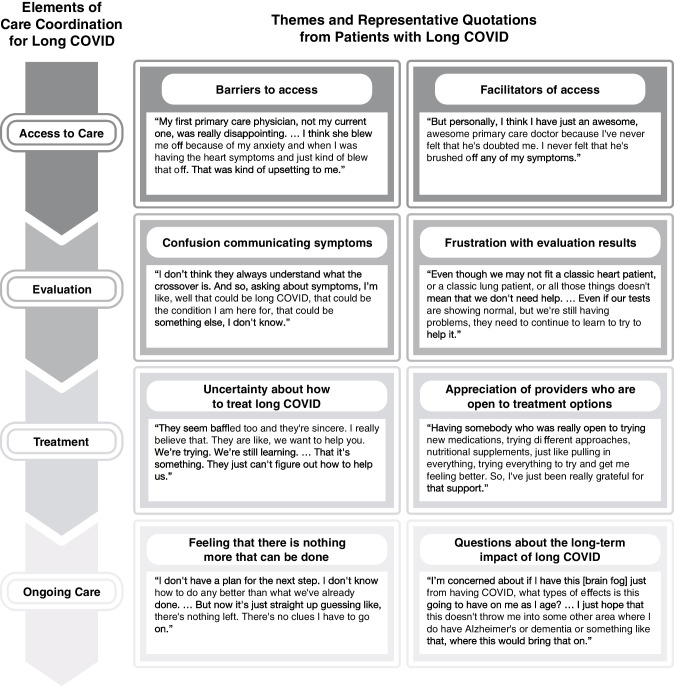
Themes and representative quotations from patients with long COVID mapped to elements of care coordination.

Interviews were conducted with 21 patients. Patient characteristics are described in Table 1. Patients had a mean age of 47.6 years (SD, 13.7; range, 19–68). Average interview length was 49.5 min. Emergent themes provided insight into patients’ experiences navigating care coordination for long COVID. These themes and representative quotations are presented in Fig. 1.

**Table 1:** Demographics and Year of First COVID Infection of Participating Patients with Long COVID

Characteristic	Participants *N*=21 *n* (%)
Sex	
Female	16 (76)
Male	5 (24)
Age	
18–44	7 (33)
45–54	7 (33)
55–64	5 (24)
65+	2 (10)
Year of first COVID infection	
2020	10 (48)
2021	6 (28)
2022	5 (24)

### Access to Care

#### Barriers to Accessing Care for Long COVID

When seeking care for long COVID symptoms, some patients described interactions with providers that impeded their access to long COVID care before they sought care at the post-COVID recovery clinic. Patients reported that their regular provider did not seem to believe them, causing them to feel like their provider was accusing them of faking or lying about their symptoms. Others described experiences in which providers attributed their symptoms to causes other than COVID, which patients perceived as a barrier to receiving care for long COVID. As one patient explained, “Sometimes people will just attribute things like this to the fact that you are anxious, or you are a depressed person, or whatever. So, I think that came into play with my first primary care physician. I think she probably thought, ‘Oh, you're just anxious. You’re just overly worried about this.’ I think that impacted my access to treatment.”

#### Facilitators of Accessing Care for Long COVID

In contrast, some patients described interacting with providers who listened to their symptoms, validated their concerns, and helped connect them to care for long COVID. As one patient described, “I never felt like what I was experiencing or like what I was sharing about how I was feeling or how I was doing was questioned or undermined or made small. … And they did what they could to figure out a solution or treatment that would help me improve or at least manage.” For some patients, the post-COVID recovery clinic provided the first of such interactions with a provider that facilitated access to care for long COVID. One patient shared, “I cried when I first talked to her [post-COVID recovery clinic provider] on the phone because she just believed me.” See Table 2 in the Appendix for additional supporting quotations.

### Evaluation

#### Confusion Communicating Symptoms

When describing their experience of being evaluated for their long COVID symptoms, patients reported complicated processes involving coordination among primary and specialty providers. Many patients reported experiencing numerous symptoms related to long COVID, for which they were referred to multiple specialists for evaluation. Several patients expressed confusion about knowing what symptoms to discuss with each provider. For example, one patient described, “My POTS [postural orthostatic tachycardia syndrome] was acting up again and I was at the cardiologist. And I didn’t think to say I’m having shortness of breath because I thought that was another issue. And I was like, I have all these symptoms and I don’t know what symptoms to tell what specialists.” This patient went on to describe that they did not know what symptoms to communicate because they did not know what some specialists did or why they were referred to them.

#### Frustration with Evaluation Results

Patients also reported frustration related to the results they received from evaluation of their long COVID symptoms. When test results came back normal, for example, several patients noted that these results did not reflect how they were feeling and should not prevent providers from further investigating their symptoms. For other patients, when evaluation resulted in a diagnosis, they reported frustration when the diagnoses were not attributed to long COVID, because this did not reflect their perspective of COVID as the catalyst of changes in their health. One patient shared, “Especially with the diagnosis of sarcoidosis, and it doesn’t have anything to do with COVID, and it’s kind of ... that one was like, ‘Okay doc you can’t tell me. This is my body, I know it best. You can’t tell me that it had nothing to do with, you know, me being ill, because I know what I was doing before, and I know what I’m capable of after.’” See Table 3 in the Appendix for additional supporting quotations.

### Treatment

#### Uncertainty About How to Treat Long COVID

When describing treatments received to address long COVID symptoms, many patients noted that they believed their providers were still learning about long COVID and were therefore uncertain how to treat this condition. Several patients also recognized their own role in learning how to treat and manage their long COVID symptoms along with their providers. As one patient explained, “It’s kind of like both of us trying to figure it out at the same time, hand in hand.” Patients also described taking ownership in learning about options for treating their long COVID symptoms, by gathering information and sharing it with their providers. One patient shared, “I researched it. And I brought forward ideas and thoughts. And these are things that these clinics are doing and looking at and what makes sense and what doesn’t.”

#### Appreciation of Providers Who Are Open to Treatment Options

Without evidence-based treatments for long COVID, many patients expressed an appreciation for providers who were open to trying new therapies. One patient explained, “Reminding me that, ‘Okay, we don’t have research to back what I’m recommending, but what do you think about trying this?’ So certainly, appreciate the transparency and the willingness to try different things that maybe we don’t really know if they’re not–they’re not proven necessarily, but a willingness to try things to help you feel better.” Several patients noted that this openness to treatment options was important to them especially when their diagnostic tests were normal, or when their providers did not understand the cause of their symptoms. See Table 4 in the Appendix for additional supporting quotations.

### Ongoing Care Concerns

#### Feeling That There Is Nothing More That Can Be Done

When reflecting about the care they had received, several patients reported feeling that there was nothing more that could be done to address their long COVID symptoms. One patient described how this information was relayed in a follow-up appointment: “The long COVID doctor that I saw, he ordered a bunch of tests and set me up with specialists. … Then I had to go back for a follow-up. I went back the second time and he basically said, ‘I’ve done everything I can. We’ve run the tests. You’re with all the specialists. There’s really nothing more I can do.’ He said, ‘We just don’t know enough about long COVID.’” In discussing follow-up to initial treatment plans, one patient described how this left them feeling like the providers were just guessing at how to address their ongoing long COVID symptoms.

#### Questions About the Long-Term Impact of Long COVID

Beyond their current evaluation and treatment, patients also expressed ongoing care concerns about the long-term effects of long COVID, especially related to aging processes such as cognitive decline, and life expectancy overall. These concerns appeared to be particularly aligned with experiencing long COVID symptoms of fatigue, shortness of breath, and brain fog. Patients expressed concerns that they were more susceptible to cognitive decline, including increased risks for Alzheimer’s disease or dementia. Patients also shared concerns that their life expectancy would be shorter due to their long COVID symptoms. One patient questioned, “Long-term what is my health going to be like? … Am I going to be more susceptible to Alzheimer’s or Parkinson’s or strokes because of clotting blood and things like that? … When I approach my 60s and my 70s, will this impact my life expectancy?” See Table 5 in the Appendix for additional supporting quotations.

## DISCUSSION

Our study reveals challenges that patients face as they navigate the healthcare system to address their symptoms. In some cases, this is due to the unknowns that still exist about the pathophysiology and treatment of this new chronic condition. In other cases, these challenges illuminate shortcomings in the ways health systems currently provide and coordinate care for complex medical needs.

Our findings corroborate evidence from other studies about the experiences of different patient populations with long COVID.^25^ In a study of healthcare workers with long COVID, participants similarly reported relief in finding providers who listened to their accounts, helped make sense of their experiences, and worked with them to diagnose and treat their conditions.^26^ Another study demonstrated the perceived importance of finding the “right” provider to help navigate care for long COVID.^27^ Healthcare workers with long COVID have also expressed frustration with aspects of care coordination, which included challenges communicating symptoms and securing referrals to specialists, as well as feeling they had to self-advocate to receive evaluation and treatment for their symptoms when their own providers did not know how to manage long COVID.^28^ Others have also uncovered perspectives that describe navigating the complexity of care for long COVID as “exhausting.”^29^ Much of this supporting evidence has been collected in the UK,^25^ which demonstrates that challenges in care coordination for long COVID exist beyond the United States’ healthcare systems. Our findings in the context of an academic medical center in the USA help expand this evidence in another setting.

Parallels can also be drawn to persons with other challenging and contested diagnoses (e.g., chronic fatigue syndrome, fibromyalgia). Similarities include difficulty crafting a coherent illness narrative, encounters with providers who minimize symptoms, extensive testing with non-diagnostic results, lengthy periods of suffering in the absence of treatment, and reliance on self-education for answers not provided by medical professionals.^30,31^ Many of these factors are determinants of the quality of care that patients receive. These similarities also reveal a common need for validation among patient populations affected by “invisible” disease.^32,33^ Our findings reiterate the need to acknowledge patient suffering, empower patients with tips for self-monitoring, and validate patients’ own research into potential therapeutic strategies, while providing patient-centered guidance on efficacy versus risk.

Our findings also reveal much about the present state of care coordination in the USA. The negative experiences relayed by our study participants expose areas where our health systems fail patients with complex illnesses, while the positive experiences reported can provide guidance for improvement. Ultimately, the gaps in care coordination highlighted in our study are indicative of several ways in which our health systems are unprepared for the influx of millions of patients with a new and poorly understood chronic condition. A critical shortage of providers knowledgeable about this condition is one cause of this unpreparedness.^1^ Primary care providers are at a loss due to the lack of evidence-based guidelines on management and treatment of long COVID. Specialists are also faced with patients reporting long COVID symptoms and, after obtaining standard diagnostic tests, may conclude that there is no organic pathology. In either situation, even if a diagnosis is made, providers remain uncertain how to treat patients.

There is an urgent need for research to inform diagnosis and treatment of long COVID and for guidelines of care to be disseminated to primary care physicians and specialists.^26^ Recommendations to improve care for patients with long COVID are beginning to emerge from research studies and professional organizations.^10–17,34–36^ However, capacity building is also needed to increase access to providers and clinics specializing in the care of long COVID. Healthcare providers who themselves have been diagnosed with long COVID have suggested the benefits of “one-stop-shop” models to decrease barriers to care for long COVID.^26^ Multidisciplinary long COVID clinics are one such option where co-located providers from multiple disciplines may increase long COVID care access for patients.^37^ Developing strategies to coordinate evidence-based care that addresses individual, community, and health system needs^21,22^ likely provides the best opportunity to manage this new chronic condition.

### Limitations

Many of the patients we interviewed had been experiencing severe symptoms for 1–2 years, which may not be a representative cross section of all patients with long COVID. Furthermore, participants were recruited from a dedicated post-COVID recovery clinic in an academic medical center. As a result, we were unable to study the perspectives of individuals living in more remote areas without access to a long COVID clinic. Additionally, we did not systematically inquire about patients’ long COVID symptoms and were therefore not able to explore how differences in symptoms may impact perspectives about seeking and receiving healthcare. Furthermore, we did not collect demographic information beyond age and gender from participants. Future research to explore issues such as care disparities for long COVID patients will be critical to improve access and provide equitable care, especially as options for specialized care for long COVID have continued to expand since the time of our study. Lastly, our understanding of long COVID continues to evolve as factors such as new variants, subsequent infections, and immunity from vaccination may impact long COVID. As our knowledge about long COVID expands, continuing research will be critical to elucidate the needs of current and future patients with long COVID.

## CONCLUSIONS

Our study revealed challenges that patients faced as they sought care for long COVID. More widespread public and medical recognition of long COVID as a serious chronic condition is needed to legitimize these patient experiences, and rigorous clinical and translational research is needed to understand the pathophysiology of long COVID and identify effective treatments. Implementing new models of care for long COVID is critical to expand accessible and equitable care for the individuals affected by this emergent chronic condition.

### Supplementary Information

Below is the link to the electronic supplementary material.

Supplementary file1 (DOCX 31.2 KB)

## Declarations:

### Prior Presentations:

This work was presented at the Society of General Internal Medicine Annual Meeting in May 2023.

### Data Sharing Statement:

Data will not be made publicly available due to concerns for participant privacy.
