# Supplementary material for: Patient Experiences Navigating Care Coordination For Long COVID: A Qualitative Study
Source: J Gen Intern Med. 2024 Feb 2;39(8):1294–300. doi: 10.1007/s11606-024-08622-z (PMC11169119; doi:10.1007/s11606-024-08622-z)
Supplement: Supplementary file 1 — Supplementary file1 (DOCX 31.2 KB) [file 11606_2024_8622_MOESM1_ESM.docx]

**APPENDICES**

**Table 2 Patient perspectives about access to care for long COVID**

| **Theme** | **Quotations** |
| --- | --- |
| Barriers to accessing care for long COVID | I want doctors to believe me. I want doctors to believe all of it, to know that this is not … I’m not making this up. That this is for real. And I know that you cannot see my joint pain, I know you can’t see my headache, I know you can’t see that my entire body is one big bruise. I know you can’t see that, but it’s real, it’s real. And so, just find something to fix it.  Well, I mean, stop trying to make us feel stupid, like we don’t know what we’re talking about. And don’t tell us to find other things to do, because we’re trying to be advocates for our own health. Try to, even if you don’t agree with us, listen to what we have to say. |
| Facilitators of accessing care for long COVID | I think the biggest thing that made an impact on me from the medical staff that I encountered was just anyone who truly believed what I was saying about how I was feeling in my own body and didn’t make assumptions about why I had symptoms, or if they were related to some other cause, or if it was irrelevant. I think sometimes just knowing that you were really heard by someone, even if they don’t agree with you, I would rather somebody hear me out and truly be empathetic with what I’m saying.  A huge relief, it was a huge relief to have somebody that was taking me seriously. |

**Table 3 Patient perspectives about evaluation for long COVID**

| **Theme** | **Quotations** |
| --- | --- |
| Confusion communicating symptoms | To listen to all the symptoms. Not make me figure out what symptoms work for the cardiologist, because sometimes they could all, I mean, there’s just so many. And so, like my shortness of breath could be cardiology, it could be pulmonology, it could be autonomic. They need to figure that out. But listen to the symptoms, and then they can decide which ones they need to work on. Because so many of mine overlap.  And she said, you know what, I want you to stop. I want you to stop writing it down, because I have all these notebooks and all these things I’m trying to figure out. She goes, it is not up to you to figure out what symptom goes with what specialist. It is our job. That was so helpful. It took pressure off me because you know, I just met with an autonomic neurologist with [healthcare system], who did a telehealth to try to figure out like with my oxygen and everything. And he was like, ‘So why are you here?’ And I’m like, ‘I don’t know. I was referred because therapists and the neurologist is saying that a lot of my symptoms are autonomic.’ I said, ‘But I don’t know what you do. So, I don’t know what symptoms to tell you.’ … But I think for them to realize that we as patients, we’re not understanding why we might have 20 symptoms. And we don’t know which doctors to treat which, so they need to be able to listen. |
| Frustration with evaluation results | Just acknowledge there is clearly something wrong, despite all the tests not showing it. Be open with people.  It took me forever to actually get them to write down a diagnosis because no one wanted to put it as COVID long. … And so I had a pulmonologist who, when I was forced to pull all the doctors notes for FMLA [Family and Medical Leave Act], saw what she was writing because I’d never thought to look. And it was not what she was saying to my face. And I understand doctor’s notes have to be written in a certain way, but it was a lot of, ‘All tests are clear, you’re just fat.’ |

**Table 4 Patient perspectives about treatment for long COVID**

| **Theme** | **Quotations** |
| --- | --- |
| Uncertainty about how to treat long COVID | I just think that we are all kind of was going through really unknown territory. We still are.  And I early on read through and found a lot of stuff that I’ve taken to my doctors and evaluated. Is this something we can look at? Does this make sense? Is there any reason why this would or wouldn’t work? Is there any harm if I try it? And I brought a lot of those ideas that I’ve scoured through to find. |
| Appreciation of providers who are open to treatment options | I am seeing a wealth of doctors who I understand don’t know how to treat it, don’t know where to start. There’s no protocol for this, but they’re willing to try. That’s one camp. I said, ‘There’s another camp that says I’ve run the traditional test, I see nothing wrong, you’re fine, it’s in your head.’ That’s another camp. I said, ‘I need to know which camp you’re in. Because if you’re in the second camp, I’m going to sever my ties with you.’ And I’m going to sever my ties with any doctors that’s in that camp.  It would be helpful just to have some time some reassurance of, ‘We hear you. We know it’s bad. And we are doing the best we can to learn how to help.’ And I get that sometimes, but not all the time. But just knowing that like my home team, and it was with [healthcare system], was amazing because they were researching on their own. And they’d come back and say, ‘Hey, I just read this study. And they’re recommending this.’ And they were always on fire to try to help, but it was so good. But then, and those people are in the trenches. They’re working with us. They’re trying to help us, but sometimes the doctors, like my family doctor when I said, ‘I have blurry vision,’ he was just like, ‘I don’t know why.’ And he doesn’t know why. But if it could be like, ‘We don’t understand it, but we’re learning.’ Just simple things like that would help encourage. |

**Table 5 Patient perspectives about ongoing care concerns**

| **Theme** | **Quotations** |
| --- | --- |
| Feeling that there is nothing more that can be done | There’s really like nothing. I guess, as far as the COVID doctor goes, I did always feel better when I left her office. The lady who coordinates all the things, sends you to all different places, the regular doctor. I felt better physically. She was positive too, and would say things like the research points to eventually you’ll get better. But there’s really, other than having the answers already, there’s nothing they can do unless they have answers.  I don’t know, the doctors that I have had through the long COVID clinic and the referrals have been really great. And every doctor has been very understanding. None of them know what to do. None of them know how to fix me. I guess, I don’t fault anybody for that. Nobody knows what in the world, nobody knows how to fix it, but I don’t know. I just want them to keep trying to find a cure, just find, just find something that fixes it. |
| Questions about the long-term impact of long COVID | Like, brain fog, is it going to, you know, with the millions of people who have experienced it post COVID, even if it’s for a short, even if it’s for a very short period of time and it resolves within two or three or four weeks, is that going to increase the risk of dementia greatly at an earlier age? … I honestly think there’s some of the researchers know more than what we’re being told. There’s a little bit of the conspiracy theorist in me.  But I guess cutting your life span shorter is a huge concern because you don’t know. Like I said, my mom is 89, my dad is 96. I got good genes. But I don’t know what difference COVID is going to make in that. I think about all the medications that I’ve taken. And still, some of the side effects, like the fatigue, and brain fog, and stuff. I guess that would pretty much be it as far as long term. I hear so many people now and even people dying like, oh boom, they got sick, and they died. I have to wonder if they had long COVID and didn’t know it. I just questioned some things like that, not out loud, but just to myself. I think more people have it than realize it or want to admit. |
